# Editorial: The culprit behind some diseases: overexpression/hyperactivity of G6PD

**DOI:** 10.3389/fphar.2024.1459741

**Published:** 2024-07-30

**Authors:** Muhammet Karaman, N. Nuray Ulusu

**Affiliations:** ^1^ Department of Molecular Biology and Genetics, Kilis 7 Aralik University, Kilis, Türkiye; ^2^ School of Medicine, Department of Medical Biochemistry, Koc University, Istanbul, Türkiye; ^3^ Koç University Research Center for Translational Medicine (KUTTAM), Istanbul, Türkiye

**Keywords:** glucose 6 phosphate dehydrogenase, Oxitative stress, NADPH, overexpression of G6PD, alzhaimer disease, cancer, metabolic disease

The pentose phosphate pathway (PPP) serves as a pivotal junction in cellular metabolism, playing a crucial role in regulating essential processes such as biosynthesis and oxidative defense. The primary factor for regulation in the activity of this pathway is glucose-6-phosphate dehydrogenase (G6PD), which is the first checkpoint enzyme of the pathway. Both hyperactivation and inactivation of this enzyme leads to metabolic imbalances. Alterations in the activity of the PPP related to hyper-activation and inactivation of the enzyme result in the occurrence of various diseases.

In this Research Topic, we aimed to focus on the critical role of the enzyme in metabolic diseases and to explore recent developments in this area. For this, we invited scientists studying diseases related to the G6PD enzyme. This collection includes important reviews, opinions, and original research articles.

Aging negatively affects mitochondrial activity, resulting in lower ATP generation and higher ROS levels. G6PD activity declines as the cell ages (Hodgkins, 2020), and the catalytic mechanism of the enzyme also changes during this period (Ulusu and Tandogan, 2006). This change causes oxidative stress in senescent cells. In this Research Topic, Yan et al. pointed out that aging-related changes in G6PD enzyme activity may result in neurodegenerative disorders. They also underline that the changing of the G6PD enzyme, which is a neuroprotective factor against the deleterious effect of endogenous ROS in the senescent period human brain, has an impact on the antioxidant system in Alzheimer’s disease. This is consistent with a previous study reported by Ulusu (2015).

G6PD is a housekeeping enzyme responsible for cell growth. During embryonic and organismal development, aberrant activation of the PPP or G6PD was observed (Wu et al., 2018b). Unlike these processes, hyperactive G6PD enzyme activity in a cell is related to tumor development (Wu et al., 2018a). There may be an upregulation of the expression of the G6PD enzyme due to many different factors such as mutation, pathogens, diseases etc. Whatever the reason, this situation can contribute to the formation or development of a tumor. Though the relationship between G6PD deficiency and infection has not been clearly understood until recent years, Sun et al. (2022) shed light on the dark side of the G6PD overexpression-infection relationship, which is one of the reasons for tumor formation/development, with a case report and literature review. Consequently, immunodeficiency resulting from G6PD deficiency causes infection. On the other side, the hyperactivity of the G6PD enzyme can be induced by both hereditary factors as well as infections. G6PD mRNA level and G6PD expression non-tumor liver tissue and tumor liver tissue infected by the Hepatitis B virus are stimulated according to non-infected liver tissues. Nonetheless, it has been demonstrated that G6PD expression level in tumor tissue is higher than in non-tumor tissue (Liu et al., 2015). In this Research Topic, Chang et al. have noted that G6PD enzyme hyperactivity is associated with another virus infection. According to their findings, human papillomavirus (HPV) causes overexpression of G6PD mRNA and G6PD protein associated with the HPV16 E6 protein in patients with cervical cancer. The same results were obtained in pcDNA-HPV16 E6 plasmid-transfected Hela cells. The relationship between infection and alterations in G6PD expression indicates that viral infections contribute to carcinogenesis by changing host G6PD expression.

Gene expression changes are among the most common reasons for G6PD overexpression. Overexpression of the G6PD enzyme may cause changes in the expression of many genes associated with different forms of cancer. In colon cancer, the G6PD enzyme is overexpressed due to upregulation of p21-activated kinase 4 (PAK4) (Zhang et al., 2017). PAK4, a member of the PAKs family, is frequently overexpressed in various tumor types and promotes carcinogenesis and progression (Wang et al., 2014) by enhancing cell growth and proliferation (Murray et al., 2010). This effect is caused by upregulating glucose metabolism as well as many other pathways. As a result, consumption of glucose and NADPH synthesis result in an increase in cancer cells. This situation is caused by increased G6PD enzyme activity as a result of PAK4’s mechanism of stopping G6PD inactivation via P53 by increasing P53 degradation of Mdm2 (Zhang et al., 2017). Another factor for G6PD overexpression is nuclear factor erythroid 2-related factor 2 (NRF2). However, its effect on G6PD expression is not straightforward but occurs through the regulation of transcription factor TAp73. This regulation occurs when NRF2 suppresses the E3 ligase PIRH2, hence prolonging the half-life of TAp73 (Zhang et al., 2022). TAp73 with enhanced half-life increases the expression of the G6PD enzyme (Du et al., 2013). Overexpression of the G6PD enzyme in cancer cells not only contributes to tumor development and formation but also causes the development of resistance to many treatment methods based on the principle of creating oxidative stress, such as chemotherapy and radiotherapy. Thus, the inhibition of enzyme activity is crucial for eliminating cancer produced by this hyperactivity. The G6PD enzyme has been a Research Topic of intense interest in the last decade due to its potential as a target of chemotherapeutic and chemo-sensitizer drug candidates for cancer treatment. Many compounds have been developed to decrease the hyperactivity of the enzyme in cancer cells, and their kinetic effects have been studied to inhibit the enzyme’s catalytic activity or prevent dimerization. The increase in the number of research articles published in the 2020s shows that the G6PD enzyme will be a common target in cancer treatment research. In this Research Topic, Xu et al. expressed that caffeine inhibits the G6PD enzyme by binding its structural NADP^+^ binding site. Caffeine reduces the formation of the dimer structure necessary for its catalytic activity. G6PD dimerization is potently related to the interaction between specific amino acids in the dimer interface. Critical residues such as T406, K403, and Y401 play a pivotal role in this process, and mutations in these amino acids can disrupt dimerization (Matte et al., 2020). In addition, the presence of NADP^+^ in the G6PD enzyme structure is another factor contributing to the dimerization process (Au et al., 1999).

Because the G6PD enzyme functions in the cell’s most crucial pathways, such as glycolysis, gluconeogenesis, PPP, and lipid metabolism, managing its activity is critical for an organism’s hemostasis. Expression of the G6PD enzyme varies depending on several regulators; it is also regulated by the control of hormones including insulin, glucocorticoids, and epidermal growth factor (EGF) in various tissues (Kletzien et al., 1994). Keran and Barker (1976) discovered that G6PD levels increase in the uterus of mature rats after 18 h following 5 µg/rat of estradiol (Keran and Barker, 1976). mRNA expression of the G6PD enzyme involved in carbohydrate and lipid metabolism downregulates with specific hormones (Pes and Dore, 2022). In the research performed on hormone-sensitive cell lines, androgen receptor (AR) was knocked down by using siRNAs in these cells. Following siRNA treatment, G6PD protein expression decreased. It has been determined that this effect is related to the motor regulation mediated by AR (Tsouko et al., 2014). A disruption in the endocrine system directly results in a change in the regulation of metabolic pathways, including PPP. Many chemicals impair endocrine function by binding hormone receptors (Schug et al., 2011; Lauretta et al., 2019), This change in the endocrine system causes a detrimental impact on human health (Diamanti-Kandarakis et al., 2009). This effect can be observed more effectively in energy metabolism and biosynthesis due to the roles of hormones in lipid, carbohydrate, and protein metabolism. There is limited research on the regulation of the G6PD enzyme by the endocrine system. In this Research Topic, Aydemir and Ulusu (2023) pointed out the endocrine-related regulation of G6PD expression. They expressed that endocrine-disrupting chemicals sourced from cosmetics, hygiene products, food and beverage packages, toys, and medical devices have impaired G6PD enzyme expression by effecting hormone metabolism. Individuals, particularly those who have G6PD deficiency, will experience metabolic and cardiovascular disorders as a result of the detrimental effects of EDC exposure throughout daily life on the G6PD enzyme.

In conclusion, this Research Topic provided detailed insights into the regulation of G6PD enzyme activity or expression, focusing on diseases related to G6PD enzyme hyperactivation. The overexpression in various disease states was discussed, with underlying causes and associated therapeutic strategies offering novel insights into treatment.
